# Surface display of proteins by Gram-negative bacterial autotransporters

**DOI:** 10.1186/1475-2859-5-22

**Published:** 2006-06-20

**Authors:** Nancy Rutherford, Michael Mourez

**Affiliations:** 1 Canada Research Chair on Bacterial Animal Diseases, Université de Montréal, Faculté de Médecine Vétérinaire, 3200 Sicotte, St-Hyacinthe, J2S 7C6, Québec, Canada

## Abstract

Expressing proteins of interest as fusions to proteins of the bacterial envelope is a powerful technique with many biotechnological and medical applications. Autotransporters have recently emerged as a good tool for bacterial surface display. These proteins are composed of an N-terminal signal peptide, followed by a passenger domain and a translocator domain that mediates the outer membrane translocation of the passenger. The natural passenger domain of autotransporters can be replaced by heterologous proteins that become displayed at the bacterial surface by the translocator domain. The simplicity and versatility of this system has made it very attractive and it has been used to display functional enzymes, vaccine antigens as well as polypeptides libraries. The recent advances in the study of the translocation mechanism of autotransporters have raised several controversial issues with implications for their use as display systems. These issues include the requirement for the displayed polypeptides to remain in a translocation-competent state in the periplasm, the requirement for specific signal sequences and "autochaperone" domains, and the influence of the genetic background of the expression host strain. It is therefore important to better understand the mechanism of translocation of autotransporters in order to employ them to their full potential. This review will focus on the recent advances in the study of the translocation mechanism of autotransporters and describe practical considerations regarding their use for bacterial surface display.

## Review

### Introduction

The display of proteins on the surface of biological organisms is of major interest in biotechnology and medicine. Several techniques have been developed to display polypeptides on the surface of viruses, yeast or bacteria [1]. Bacterial surface display has found many applications in recent years [1,2]. Displaying single or multiple epitopes, as well as complete proteins, on the bacterial surface can for instance yield recombinant vaccines that can possibly be taken orally [3]. Such recombinant bacteria can also be used for the production of antigens or antibodies necessary in the design of diagnostic tools. In another application, bacteria can be engineered to display functional recombinant enzymes at their surfaces and can thus be used as "biofactories", with many biotechnological applications [4-6]. In a variation of this principle, the use of bacterial hosts that can resist various types of pollution and have been engineered to display proteins able to fix heavy metals has been applied to the design of new bioremediation approaches [7]. Lastly, bacterial surface display has also been used in the high throughput screening of peptides and enzymes libraries [8,9].

Surface display consists in genetically fusing a protein of interest to another protein that will allow its presentation at the surface of an organism [1]. Bacterial display thus exploits a mechanism that mediates the export of the protein of interest from the cytoplasm to the surface [2]. Both Gram-positive and Gram-negative bacteria might be used for surface display [10]. Gram-negative bacteria, however, possess two membranes that proteins need to cross in order to reach the extracellular milieu and these bacteria resort to different secretion systems to target proteins to their surface. Most bacterial proteins that are exported across the inner membrane use either the general secretion (*sec*) or the twin arginine translocation (*tat*) pathways [11,12]. From the periplasm, integral outer membrane proteins (OMP) are then inserted, either spontaneously, or using a specialized machinery comprising the protein Omp85 [13] and other envelope proteins [14]. LamB [15] and OmpA [16] were the first OMPs to be used for bacterial surface display in *Escherichia coli *but since then, many more have been employed, including PhoE [17], OmpC [18], TraT [19], FhuA [20], or intimin [21]. Some surface exposed outer membrane lipoproteins have also been used for bacteria surface display, such as the ice nucleation protein of *Pseudomonas syringae *[22]. From precursors in the periplasm, Gram-negative bacteria also assemble complex structures at their surfaces, such as flagella, pili or S-layers, all of which have been used for display of proteins or peptides [23-25]. Lastly, several dedicated protein secretion system in Gram-negative bacteria allow the surface anchoring of the proteins they transport [26]. Some of these systems have been used for the display of proteins. This is the case, for instance, with the type II secretion system. The type II secretion system, also called the main terminal branch of the *sec *pathway, permits the translocation in the extracellular medium of proteins exported in the periplasm by the *sec *or *tat *pathway [27]. This complex machinery comprises at least a dozen proteins situated in the inner membrane, the periplasm and the outer membrane. One substrate of a type II secretion system, the enzyme pullulanase from *Klebsiella oxytoca *was used to display the periplasmic enzyme β-lactamase on the surface of *E. coli *[28]. The type III secretion system has also been used as a display system. This system is genetically and structurally analogous to the flagellum and comprises up to 20 different proteins found in each layer of the bacterial envelope [29]. The system functions as a molecular syringe delivering bacterial virulence proteins from the bacterial cytoplasm directly into the extracellular milieu or the cytosol of plant or animal cells. The main structural component of the type III secretion system of *E. coli*, EspA, has recently been used as a display system for short polypeptides [30]. But the most widely used secretion system for bacterial surface display has been the type V secretion system and more specifically the subgroup of monomeric autotransporters (AT) [31].

### Autotransporters

The first AT discovered was the *Neisseria gonorrhoeae *IgA protease [32] and the term 'autotransporters' was coined when it was realized that more proteins exhibited the similar striking feature of being translocated from the periplasm across the outer membrane as a single polypeptide that contained all the information necessary for secretion [33]. Since then, many AT have been identified. Most are involved in the virulence of bacterial pathogens and take part in various processes such as adhesion, aggregation, invasion, biofilm formation, serum resistance and cytotoxicity [31]. AT belong to the type V secretion system of which they constitute the type Va branch [31]. Similar proteins, initially grouped with the IgA proteases, were subsequently found to require trimerization in order to promote their secretion [34-36]. Hence, these proteins form the trimeric autotransporters group [37], or type Vc branch of the type V secretion system [31]. Lastly, the two-partner secretion system (TPS) is closely related to AT. Whereas AT are single polypeptides that contain two domains, one which is secreted across the outer membrane and another one which mediates this translocation step, in TPS systems the two domains are synthesized as two independent polypeptides [38]. TPS constitute the type Vb branch of the type V secretion system [31]. TPS and trimeric autotransporters have seldom been used for bacterial surface display and mostly for the purpose of translocation studies [34]. They will therefore not be discussed further.

**Figure 1:**
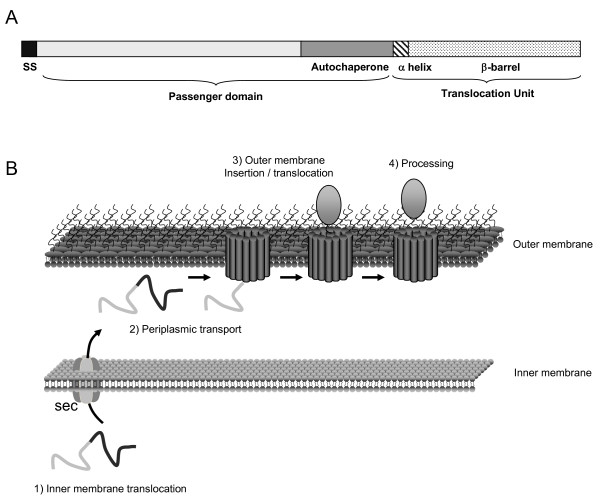
**Organization and biogenesis of autotransporters**. A. The typical organization of an autotransporter (AT) comprising an N-terminal signal sequence (SS), a passenger domain and a translocation unit (TU). The passenger domain includes an N-terminal part that bears the activity of the AT and a C-terminal domain, called the autochaperone domain, which is important for efficient translocation across the outer membrane. The TU also has two distinct domains; the N-terminal region is structured as an α-helix, whereas the C-terminal region is structured as a β-barrel. B. The biogenesis of an AT has four main steps: translocation across the inner membrane, periplasmic transport, insertion into and translocation across the outer membrane and, lastly, processing of the passenger domain.

Autotransporters share a distinctive organization in three main domains (Figure 1A, [32]). At their N terminus they have a *sec*-dependent signal peptide followed by a passenger domain comprising the functional part of the protein. This passenger domain is translocated through the outer membrane thanks to a C-terminal region called the translocation unit (TU). TU are composed of two sub domains: the C-terminal 250 to 300 aminoacids that are predicted to form a β-barrel inserted in the outer membrane [39,40] and, immediately before that, a 25 aminoacids linker region predicted to adopt an α-helical conformation [41,42]. The extracellular part of the AT, the passenger domain, has also two sub domains: an N-terminal domain that bears the activity of the AT and a C-terminal domain called the autochaperone domain [43]. This autochaperone domain is dispensable for translocation but increases its efficiency, either by stabilizing the β-barrel [44], or by promoting the folding of the passenger domain [43,45,46].

The mechanism of translocation is thought to be conserved among the different AT and to contain four steps: (i) inner membrane translocation, (ii) periplasmic transport, (iii) insertion into and translocation across the outer membrane, and (iv) processing of the passenger domain (Figure 1B).

#### a) Translocation across the inner membrane

Autotransporters exhibit an N-terminal sequence bearing most of the typical features of *sec*-dependent signal peptides [11]. But some AT, such as the group of serine proteases autotransporters of *Enterobacteriaceae *(SPATEs), have an unusually long signal peptide [47]. The long peptide signal sequences have a conserved domain structure, bearing an N-terminal extension which has a relatively conserved sequence, whereas the other domains of the signal sequence do not. A similar long signal is also found in some trimeric autotransporters, such as the *Haemophilus influenzae *Hsf and Hia adhesins [48,49], and some TPS systems, such as the *H. influenzae *HMW adhesins and the *Bordetella pertussis *filamentous haemagglutinin [50,51].

Despite this puzzling characteristic, little attention was devoted to the mechanism of transport across the inner membrane. Two main targeting pathways to the *sec *machinery co-exist in bacteria: The SecB protein targets bacterial protein post-translationally whereas the signal recognition particle (SRP) targets bacterial proteins co-translationally to the *sec *translocon, via its membrane receptor FtsY [52]. A report showed that the targeting to the *sec *machinery of the *E. coli *haemoglobin protease, which possesses an unusually long signal peptide, was mediated by SRP [53]. In this report, it was shown that the extended signal sequence can strongly be cross-linked to SRP *in vitro *[53]. Depletion of the SRP pathway, however, had only a moderate but significant effect *in vivo *on the efficacy of the protease secretion [53]. In another study, when the extension of the unusually long signal sequence of the *E. coli *AT EspP was removed the resulting truncated signal sequence allowed interaction with SRP [54]. These results therefore suggested that AT, and possibly even those without unusual long signal sequence, might be crossing the inner membrane co-translationally, presumably to avoid adverse effects the cytoplasm might exert on their passenger domain. But another report challenged this view by showing that the secretion of the *Shigella flexneri *AT IcsA, which also bears an unusually long signal peptide, is independent of the SRP pathway [55]. This study did confirm, however, that IcsA crosses the membrane via the *sec *machinery [55]. Furthermore, it was reported that the extension of the unusually long signal sequence was not required for crossing the inner membrane [56]. More recently, it was shown that the full length signal sequence of EspP does not interact with SRP but instead recruits SecB [57], despite the fact that, as mentioned above, the same group reported that the signal sequence without the extension does interact with SRP [54]. It was therefore hypothesized that the extension might actually bypass SRP. Lastly, it could be noted that the co-translational secretion model across the inner membrane is hardly compatible with the fact that the *E. coli *AT adhesin involved in diffuse adherence (AIDA-I) is glycosylated by a cytoplasmic heptosyl-transferase [58]

In summary, the influence of the N-terminal extension on the targeting to the *sec *machinery and the co-translational versus post-translational nature of the targeting remain controversial and will require further studies. Regardless of the targeting mechanism, however, one study showed that the role of the extension might be to cause a slow down of the release from the *sec *machinery after translocation in the periplasm [56]. It was proposed that this very unusual property for a signal peptide might be required to prevent misfolding of the passenger domain in the periplasm [56]. This result awaits confirmation from other independent studies.

#### b) Periplasmic transport

There are many lines of evidence suggesting that, after crossing the inner membrane, the protein exists as a periplasmic intermediate. Indeed, as discussed below, various studies have shown that passenger domains can fold in the periplasm and that this process is influenced by the genetic factors of the host affecting the periplasmic environment. These experiments therefore suggest that the passenger domain of an AT has access to the periplasmic environment before its translocation across the outer membrane. Furthermore, a periplasmic intermediate was isolated in the case of IcsA [59].

There is, however, a nagging controversy over the extent of folding that the passenger domain of an AT undergoes while in the periplasm. Most studies addressing the question of passenger domain folding in the periplasm have resorted to replacing original passenger domains with heterologous proteins [60-69]. The earliest experiments were performed using the B subunit of the cholera toxin (CtxB) fused to the TU of the *Neisseria *IgA protease. CtxB contains cysteines that can become disulphide bonded in the periplasm. It was initially shown that the outer membrane translocation of CtxB is affected when it is able to form disulphide bonds, and that the protein was blocked in the periplasm [60,61]. Indeed, CtxB mutants without cysteine were translocated more efficiently, and a similar effect was observed when adding 2-mercaptoethanol in the growth medium or when using *dsbA *mutant strains, conditions that prevent the formation of disulphide bonds in the periplasm [65]. Disulphide bond formation is a slow step on the path of protein folding and the presence of such bonds is often used as a marker for folding. These experiments were therefore interpreted as indicative of an incompatibility between periplasmic folding and outer membrane translocation in AT. Thus, it was concluded that the native passenger domains of AT must remain unfolded in the periplasm. In other experiments, PhoA, a periplasmic protein containing cysteines, was fused to the TU of IcsA [63]. The translocation of PhoA across the bacterial outer membrane was incomplete and could be increased when it was not allowed to form disulphide bonds in the periplasm, confirming the results observed with CtxB.

Recent studies, however, showed that single-chain antibody fragments (ScFv) fused to the TU of the *Neisseria *IgA protease can be found in an active conformation on the bacterial surface [66,67]. Disulphide bond formation is necessary for most ScFv to adopt a correctly folded conformation and it was shown that the disulphide bond could be formed in the periplasm. Thus it was concluded that the protein was folded prior to translocation, contradicting the previous hypothesis. More experiments soon followed, suggesting that polypeptides at least partially folded could indeed be translocated across the outer membrane. For instance, the native passenger domain of IcsA was shown to contain a disulphide bond in the periplasm prior to translocation [59]. Lastly, another study using fusions between CtxB and the TU of EspP showed that translocation of a passenger domain at least partially folded was possible [69]. From these studies, one can postulate that the native passenger domains of AT might fold, at least partly, in the periplasm.

All those experiments were performed using disulphide bond formation as an indicator of folding, and by manipulating the ability to form those bonds in order to assess the effect of folding on translocation. The absence or presence of disulphide bonds, however, can not always correlate with the extent of folding of a protein [70,71]. The number of aminoacids separating two bridged cysteines as well as the propensity of the resulting loop to adopt a structure will greatly influence the resulting shape of a protein bearing a disulphide bridge. Furthermore, manipulating the ability to form disulphide bonds by mutating the cysteines of passenger domains or by changing the periplasmic redox environment can have uncontrolled effects on the protein via the induction of bacterial stress responses [72]. This can complicate the interpretations of studies resorting to the observation of the effects of disulphide bridges when their goal is to evaluate the impact of folding on translocation. Indeed, it has been reported that the precursor of OmpA can be translocated through the *sec *machinery even in the presence of a disulphide bridge [73], although it is accepted that the *sec *machinery exports unfolded polypeptides. A recent study using MalE, a periplasmic protein without cysteine, fused to the TU of AIDA-I showed that the folding of the passenger domain interferes with translocation by using MalE variants with intrinsic folding defects [74].

Putting the published observations together, it seems likely that the native passenger of AT can undergo some folding in the periplasm but that the nature or the extent of the folding of the native passenger domains is probably limited. How can this controlled folding can happen in the periplasm, and is there an involvement of periplasmic chaperones in this process? These questions have hardly been addressed. A recent study showed that the peptidyl-prolyl isomerase FkpA might play a role in the secretion of a ScFv fused to the TU of the *Neisseria *IgA protease [67]. Furthermore, as described above, the unusually long signal sequence of some AT might delay the dissociation from the *sec *machinery, and therefore control periplasmic folding [57].

The influence of many more periplasmic folding catalysts, such as SurA or PpiA for instance [75], remain to be investigated. It will however be difficult to discriminate between a contribution of these factors to the folding of the TU and their role in the folding of the passenger domain.

#### c) Insertion into and translocation across the outer membrane

Many different models have been proposed to describe the insertion of an AT in the outer membrane [31], based on the structural studies of the TU and passenger domains, and on the observations regarding periplasmic folding of the passenger domain described above. The minimal requirement for translocation resides in the TU, and in some AT the limits of the TU were experimentally mapped [39,76]. The TU structure was predicted to consist of an α-helix and a β-barrel [40-42]. Recently, the crystal structure of the TU of the *N. meningitidis *AT NalP was obtained [77], confirming earlier studies. The structure consists in a twelve-stranded β-barrel and an α-helix inserted in the β-barrel lumen with the N terminus of the α-helix, which links to the passenger domain, localized at the extracellular side of the outer membrane. It is interesting to note that previous predictions have sometimes put the α-helix partly inserted in the membrane rather than in the lumen of the barrel [41]. The structure of the TU of NalP shows numerous salt bridges between the helix and the lumen of the barrel. In addition, it was observed that barrel can form a pore, the conductance of which is increased when the helix is absent, a finding that is consistent with the structure of the helix partially blocking the pore. These arguments tend to strengthen the validity of the structure of the TU of NalP. It should be noted, however, first that the protein used to determine the structure was obtained from inclusion bodies refolded *in vitro*, a process that could influence the final structure, and second that the limits of the TU of NalP were never experimentally tested, i.e. it is not known whether the whole TU is present in the structure solved.

Nevertheless, in the solved structure the dimension of the pore of the β-barrel can be estimated at about 10 by 12.5 angströms and two conductance states of 0.15 or 1.3 nS were recorded with the pure protein reconstituted in planar lipid bilayers [77]. Other studies have shown that the TU of an AT can form pores in lipid bilayers [78], or in liposomes [79,80]. These studies indicated the existence of bigger-sized pores: the conductance of the TU of the *B. pertussis *AT BrkA was estimated at about 3 nS [78], and the size of the TU of the *Neisseria *IgA protease and of the *Pseudomonas *PalA were estimated at 2 nm [79,80].

These sizes have to be put in relation to the known structures of some native AT passenger domains, and to the studies on the folding state of the passenger domain in the periplasm discussed above. Natural passenger domains of AT are always polypeptides of at least 300 aminoacids and the analysis of the sequences of many natural passenger domains of AT suggested a preference towards β-strands [81]. This prediction is confirmed by the structures of the two AT passenger domains solved to date, that of *B. pertussis *pertactin [82], and that of *E. coli *haemoglobin protease [83]. Both structures share a similar fold consisting of an extended right-handed parallel β-helix. This helix has a cylindrical shape with a diameter of 20 to 30 angströms and a length of 100 to 140 angströms. In both structures, however, additional protruding loops or whole domains considerably extend the diameter of the cylinder to sizes up to 80 angströms.

As described above, the studies pertaining to the folding state of the passenger domain have reached different conclusions and have been performed with heterologous passenger domains. Some studies found that passenger domains of even very small sizes, such as the 62 aminoacids long aprotinin, could not be translocated folded [64], other studies with passenger domains of 13 kDa (CtxB) or 30 kDa (ScFv) suggested that folded protein domains can be translocated [67,69]. As stated above, the nature or the extent of folding compatible with translocation is therefore still unclear. In this respect, it is striking to note the conservation of the typical β-helical fold in the backbone of passenger domains of AT. It is possible that this structure is uniquely adapted to the translocation mechanism.

**Figure 2:**
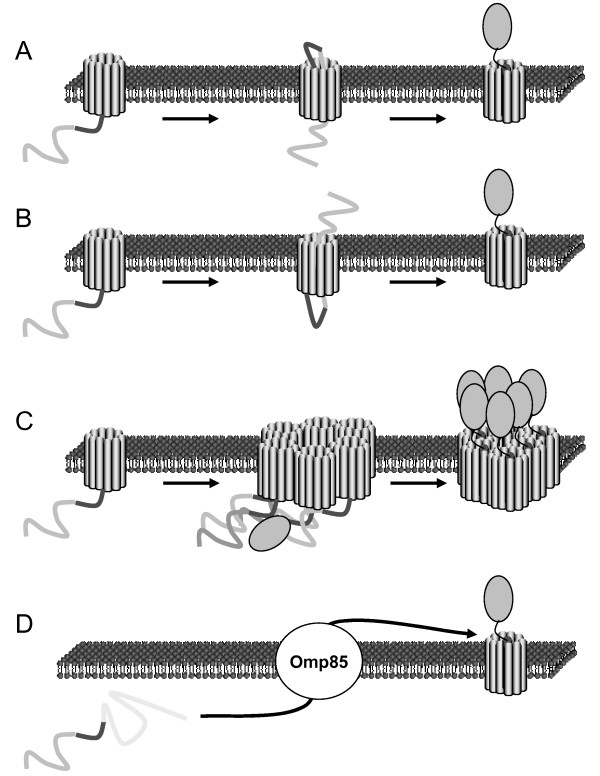
**Mechanistic models of outer membrane translocation by autotransporters**. A. Hairpin model: In this model the C-terminal part of the passenger domain inserts itself as an unfolded polypeptide in the barrel of the TU, forming a hairpin. The extracellular folding of the autochaperone domain then pulls the remainder of the passenger domain. B. Threading model. This model is similar to the hairpin model but postulates that the N-terminal part of the passenger domain is inserted first, without hairpin. C. Multimeric model. In this model, multiple TUs are assembling in an oligomer forming a big central pore. Folded and unfolded polypeptides could then cross the outer membrane through this pore. D. Omp85 model. In this model, a number of periplasmic and outer membrane proteins organized around Omp85 are involved in the insertion of the TU and also the translocation across the outer membrane of the passenger domain.

Based on all these data, different models have been put forward (Figure 2). In some models, the passenger domain is thought to cross the outer membrane mostly unfolded, through the lumen of the barrel. The small size of the observed structure of the TU of NalP is consistent with this hypothesis. According to this hypothesis, the folding of the translocating polypeptide once it reaches the extracellular milieu could also provide the energy for translocation. Two models have been proposed based on this hypothesis. One model suggests that the passenger domain is translocated starting with its C terminus, the autochaperone domain. This would result in the formation of a hairpin structure and thus the model was called "hairpin model" ([32], Figure 2A). The NalP pore still remains barely sufficient to accommodate two unfolded polypeptides simultaneously. An alternative model suggests that the passenger domain is translocated starting with its N terminus. This model, called the "threading model" ([77], Figure 2B), might alleviate the steric problem of the hairpin model because only one polypeptide would be in the pore during translocation. However, this model seems unlikely because it implies the recognition of the N terminus by the TU, whereas unrelated heterologous passenger domain can also be translocated.

None of these models can take into account the translocation of even partially folded polypeptides because of the narrow lumen of one β-barrel. Another model, a "multimeric model" (Figure 2C), was proposed, based on the observation that TUs of AT could form ring-like structures visualized by electron microscopy [79]. The size of the central cavity is large enough to allow translocation of small folded proteins or protein domains. The multimeric model therefore solves the steric problems of the hairpin or threading models. This model, however, is inconsistent with the structure of the TU of NalP for which no oligomer was observed [77]. It is also inconsistent with the observation that the α-helix, which is connected to the passenger domain, is located inside the lumen of the β-barrel and not outside, as the multimeric model would predict [77]. Furthermore, if passenger domains are translocated folded, it is unclear what the energy source for the translocation would be. Lastly, two studies have recently demonstrated that the TU of AIDA-I and EspP are not multimeric [69,84]. The oligomerization of the TU does not therefore seem to be a common feature of AT.

As can be deduced from above, all the previous models entail important steric and/or mechanistic problems. An alternative model, called the "Omp85 model" ([77], Figure 2D), was therefore proposed. This model hypothesize that translocation of AT involves Omp85, the protein that promotes the insertion of integral outer membrane proteins [13]. Since Omp85 is implicated in the insertion of outer membrane proteins, some of which contain extracellular loops representing up to half of the protein, it was suggested that the same machinery could be involved in the translocation of passenger domains. With this hypothesis it is conceivable that some folded domains might be accommodated and others rejected from translocation. It is however difficult to differentiate the contribution of Omp85 in the insertion of the TU and in the translocation of the passenger domain.

In conclusion, the "Omp85 model" remains untested but it is the model that seems to be the most favoured [69,77], probably because it is the only one that could possibly account for all the observations collected to date. Several issues are still unresolved, though, and will need to be addressed in future studies. First, the role of Omp85 in the translocation of passenger domains will have to be rigorously tested. In this respect, it will be important to determine if the translocation of passenger domain and the insertion of the TU are performed in two separate steps or in one single step. A related second question will be to evaluate if the passenger domain uses the TU and/or Omp85 as a translocation conduit. Trapping an intermediate in the translocation of an AT would be instrumental in shedding some much needed light on these questions.

#### d) Modification and processing of the passenger domain

In many AT, the passenger domain is cleaved after translocation and the passenger domain is released in the extracellular milieu. This is for instance the case of the SPATEs, many of which act as extracellular toxins [85]. In some AT, a part of the passenger domain can be cleaved and released as a regulation mechanism. In this manner, the amount of passenger domain found on the bacterial surface, and hence the activity it confers, can be monitored. This is for instance the case for the *H. influenzae *adhesin Hap [86]. It is interesting to note in that case that the cleavage can be influenced by host factors such as the secretory leukocyte protease inhibitor [87]. A regulatory cleavage was also shown to participate in the polar localization of IcsA [88]. In other AT, the passenger domain is cleaved but remains non-covalently associated to the outer membrane and the release of the cleaved fragment is not detected under physiological conditions. This is for instance the case of the *E. coli *AIDA-I adhesin [89]. The role of the cleavage in those cases remains mysterious. Lastly, some AT are not cleaved after translocation, such as the *E. coli *TibA adhesin [90].

In many cases, the cleavage in the passenger domain is thought to be autocatalytic. This has been peculiarly studied in the case of AT bearing a serine protease catalytic site. In the case of the Hap adhesin, for example, the serine protease catalytic site was shown to act *in trans *[91]. To our knowledge, autoproteolysis *in cis *has not yet been demonstrated. In some other cases, the cleavage might be performed by an accessory outer membrane protease. This is for instance the case with the protease SopA of *S. flexneri *that cleaves IcsA [92], or with NalP, which is involved in the processing of the *Neisseria *IgA protease [93]. In some cleaved AT, however, no protease catalytic site has been identified and no accessory protein has been found responsible for their cleavage post-translocation. This is the case, for example, of the *E. coli *AIDA-I adhesin [94]. Lastly, it should be noted that in some AT the passenger domain can be lipidated [93,95,96] or glycosylated [58,90,97].

### Using autotransporters as a tool for surface display

As described above, bacterial surface display has found many uses in the past few years. When it was realized that they could efficiently present heterologous polypeptides instead of native passenger domains to the bacterial surface [60], AT quickly became interesting tools for bacterial display. As shown in Table 1, they have successfully been used for various purposes, including display of antigens for vaccine purposes, display of enzymes and screening of peptide libraries. Indeed, many features make AT an attractive display system:

**Table 1:** Heterologous polypeptides fused to autotransporters and their surface display applications

**Displayed polypeptides**			**Translocator**^c^	**Application**	**References**
Name and origin	Size^a^	Remarks^b^			

CtxB, *Vibrio cholerae*	13 kDa	1 disulphide, Secreted	EspP (*E.coli*), 450 aas/711 aas	Translocation studies	[69]
StxB, *Shigella dysenteriae*	7.7 kDa	1 disulphide, Secreted	MisL (*S. enterica*), 505 aas/955 aas	Vaccination	[100]
Epitope, *Plasmodium falciparum*	32 aas	No disulphide		Vaccination	[98, 100]
Aprotinin, bovine	62 aas	3 disulphides, Secreted	AIDA-I (*E. coli*), 440 aas/1287 aas	Translocation studies	[64]
OspG, *Borellia burgdorferi*	22 kDa			Translocation studies	[84]
Sorbitol dehydrogenase, *Rhodobacter sphaeroides*	29 kDa	No disulphide, Cytoplasmic		Enzyme activity	[5]
UreA, *Helicobacter pylori*	211 aas	No disulphide, Cytoplasmic		Vaccination	[99]
Epitope, *Yersinia enterocolytica*	16 aas	No disulphide		Vaccination	[3]
Fragment of invasin, *Mycobacterium tuberculosis*	58 aas	No disulphide		Study of displayed protein	[113]
Adrenodoxin, bovine	14.4 kDa	No disulphide, Secreted		Enzyme activity	[6]
β- lactamase, bacterial	286 aas	1 disulphide, Periplasmic		Translocation studies	[62]
LTB, *Escherichia coli*	11.6 kDa	1 disulphide, Secreted		Translocation studies	[68]
CtxB, *Vibrio cholerae*	13 kDa	1 disulphide, Secreted		Translocation studies	[114]
Synthetic protease inhibitor	15 aas	No disulphide		Random screening	[110]
MalE, *Escherichia coli*	366 aas	No disulphide, Periplasmic		Translocation studies	[74]
Lipase, *Bacillus subtilis*	181 aas	Secreted	EstA (*P. aeruginosa*), 686 aas/686 aas	Enzyme activity	[101]
Lipase, *Fusarium solani*	214 aas	Secreted		Enzyme activity	[101]
Lipase, *Serratia marcescens*	613 aas	Secreted		Enzyme activity	[101]
β- lactamase, bacterial	286 aas	1 disulphide, Periplasmic	EstA (*P. putida*), 325 aas/686 aas	Translocation studies	[108]
FimH lectin domain, *Escherichia coli*	177 aas	1 disulphide, Secreted,	Ag43 (*E. coli*), 891 aas/1039 aas	Translocation studies	[106]
Various epitopes	12–14 aas	No disulphide	Ag43 (*E. coli*), 1039 aas/1039 aas^d^	Vaccination	[106]
MalE, *Escherichia coli*	366 aas	No disulphide, Periplasmic	IcsA (*S. flexneri*), 338 aas/1102 aas	Translocation studies	[63]
PhoA, *Escherichia coli*	471 aas	2 disulphides, Periplasmic		Translocation studies	[63]
Pseudo-azurin, *Alcaligenes faecalis*	123 aas	No disulphide, Periplasmic	Ssp (*S. marcescens*), 356 aas/1045 aas	Translocation studies	[112]
Fos and Jun leucine zipper domains	8 kDa	No disulphide, Intracellular	IgA protease (*N. gonorrhoeae*)	Translocation studies	[115]
Single-chain antibody (ScFv)	30 kDa	2 disulphides, Secreted	440 aas/1505 aas	Translocation studies	[66]
Single-chain antibody (ScFv)	30 kDa	2 disulphides, Secreted		Drug delivery	[116]
CtxB, *Vibrio cholerae*	13 kDa	1 disulphide, Periplasmic		Translocation studies	[60]
β- lactamase/trypsin inhibitor	314 aas	4 disulphide, Periplasmic		Random screening	[9]
Metallothionein, murine	7 kDa	No disulphide, Intracellular		Enzyme activity	[7]

^a^: sizes are given as molecular weights (in Daltons) or as a number of aminoacids (aas); ^b^: When known, the number of disulphide bridges normally found in the displayed polypeptides are indicated, as well as an indication of their original cellular localization. ^c^: Unless otherwise indicated, all fusions were made to a C-terminal fragment of an AT, the name of the AT is provided, along with the bacterial species from which it originates and the lengths of the fragment and full length protein. ^d^: The polypeptides were inserted as "sandwich fusions" in the full-length sequence of Ag43.

#### - Display of polypeptides of various sizes

AT can display small polypeptides, such as 10 to 15 aminoacids-long antigens for vaccines [68,98-100] or full size proteins of up to 613 aminoacids, as in the case of the display of the *Serratia marcescens *lipase fused to EstA [101]. In addition, polypeptides displayed by AT were shown to be able to oligomerize, a characteristic that allow dimeric enzymes to be active [5,6], and which could possibly be applied to display protein hetero-oligomers. This versatility contrasts with other systems that are restricted to polypeptides of less than 100 aminoacids because of toxicity issues [104] or structural constraints, such as what has been reported with display in fimbriae subunits [102] or with the outer membrane protein LamB [103]. It should be noted, however, that other systems have been shown to display quite large proteins, such as the ice nucleation protein or a phage display system, which were shown to display protein of 424 aminoacids [22] or 471 aminoacids [105], respectively.

#### - Display of polypeptides as N-terminal fusions

In many bacterial display systems, such as flagellin or pilin [102], some carrier proteins need their N and C termini to remain free in order to interact correctly with other proteins promoting their export. Additionally, some carrier integral outer membrane proteins have both N and C termini located in the periplasm [103]. In both cases, the display strategy therefore requires to insert foreign proteins in permissive loops of these carriers, thus creating a so-called "sandwich fusion". Finding and characterizing those sites is time-consuming and the use of one site versus another might be insert-specific [24]. By contrast, the polypeptides displayed by AT are fused to the N-terminus of the TU, a situation which alleviate some of these issues (although there are also reports of sandwich fusions with AT [106]). It should be noted, however, that this system is not amenable to the display of eukaryotic cDNA libraries. Other bacterial display systems can be used for the display of polypeptides as C-terminal fusions, such as the *E. coli *outer membrane protein intimin [21] or the chimera resulting from the fusion between parts of the *E. coli *outer membrane proteins Lpp and OmpA [107].

#### - High level of expression and activity with little toxicity

In many cases it was noted that AT display allowed high level of heterologous polypeptides presentation. The enzyme β-lactamase, for instance, was expressed at higher amounts when fused to the TU of the *P. putida *AT EstA, than when it was fused to the ice nucleation protein [108]. The amount of the enzyme sorbitol dehydrogenase or of bovine adrenodoxin fused to the TU of AIDA-I was also found to be in excess over the major outer membrane porins, and estimated to be about 100,000 molecules per cell [5,109]. A similar result was found with the display in *E. coli *of CtxB fused to the TU of *Neisseria *IgA protease, which was estimated to be about 50,000 copies per cell [39]. Such high level of expression is desirable for some applications, such as maximizing enzyme activity or using fluorescence assisted cell sorting (FACS) [110], but it can be a drawback when trying to screen libraries of binding proteins, because low affinity clones will be enriched.

Despite such high levels of expression there is often very little toxicity reported when using AT for display [66,108]. In cases where some toxicity was reported, it was not correlated with the size of displayed polypeptides [101]. It should be noted, however, that high levels of toxicity have sometimes been reported [9], and that it is also often noted that reducing the temperature of growth enhances the expression level or the activity of the displayed polypeptides [62,108].

A drawback associated with the anchoring of enzymes to the bacterial surface, however, is that the complexity of the surface of bacteria, including their surface polysaccharides, can become a barrier to the accessibility of the displayed enzymes to its substrates or impose structural constraints resulting in lower specific activity [1]. In this regard, AT have been shown to retain a significant portion of the activity of displayed enzymes. It was for instance observed that the specific activity of the enzyme β-lactamase displayed when fused to the TU of AIDA-I was 20% of that of purified free enzyme [62]. A similar finding was reported with the display of sorbitol dehydrogenase [5]. Recently, instead of fusing the TU of AT to heterologous passenger domains, polypeptides to be displayed have also been fused to all or most of the passenger domains. This was shown with the display of the adhesin FimH fused to Ag43 [106] and with the display of lipases fused to EstA [101]. In those cases, the passenger domain could possibly act as a spacer that could keep the displayed polypeptide away from the membrane and thus increase specific activity, but this was not evaluated.

#### - Versatility

It is striking to note that one construct based on an AT from one bacterial species can be used in different other bacterial species. It has been for instance shown that the same construct was displayed in *E. coli *and *Salmonella *or *Shigella *[60,61,63,106], or in *E. coli *and *Ralstonia eutropha *[7]. This is probably due to the fact that AT are found in many different Gram-negative bacteria and that the overall topology of TU seems much conserved. It should be noted, however, that the use of the TU of the *Helicobacter pylori *AT VacA seems more restricted, as it was far less well expressed in *E. coli *[111]

Because of all these features, AT appears to be peculiarly well adapted tools for bacterial surface display. However, as detailed above, the recent progress made in our understanding of the structure and function of AT highlights important details to be considered when contemplating the use of AT as display systems:

#### - Nature of the passenger domain

In various studies where the same translocator was used in the same strain to display different heterologous passengers, it was noted that the fusions achieved very different yields of expression [62,101]. As described above, the presence of cysteines able to form disulphide bonds has often been reported to interfere with translocation [60,63,65], and, more generally, the periplasmic folding of a protein could interfere with its translocation [74]. If the protein to be displayed contains cysteines, their location in the protein should be considered. If cysteines are close in the sequence of the protein, it is possible that the formation of a disulphide bond does not require the protein to adopt an extended tertiary structure and therefore does not affect translocation. However, translocation often seems to be enhanced when the protein does not contain any disulphide bond. Thus, one way to enhance the translocation of cysteine-containing protein is to change these residues by site-directed mutagenesis [60]. If the cysteines cannot be replaced by other aminoacids, the use of a *dsbA*^- ^strain or the addition of 2-mercaptoethanol in the growth medium has often been shown to enhance the amount of proteins requiring a disulphide bridge that can be displayed [7,61-63,65,108]. It should be noted, however, that aprotinin, a protein that folds quickly and which is stabilized by the presence of disulphide bonds, could not be translocated by AIDA-I, even in a *dsbA*^- ^background [64]. Translocation was only achieved in the presence of 2-mercaptoethanol [64]. Preventing disulphide bond formation might however prevent the normal folding of the displayed protein or cause some toxicity. Indeed, when β-lactamase fused to the TU of AIDA-I was expressed in a *dsbA*^- ^background it was significantly more expressed, but there was twice more enzymatic activity in the a *dsbA*^+ ^background [62]. Similarly, the absence of DsbA increased the amount of ScFv that could be displayed when fused to the TU of *Neisseria *IgA protease, but the ScFv was only active when expressed in a *dsbA*^+ ^context [66]. Lastly, other features of the polypeptide to be displayed can cause potential problems, such as hydrophobicity or the presence of charges. The N terminus of UreA was for instance deleted in order to be displayed in fusion to the TU of AIDA-I because it contained lysine residues, which might affect the secretion through the *sec *machinery [99].

#### - Nature of the signal sequence

Three approaches have been employed in this regard. A first approach is the use of the native *sec*-dependent signal sequence of the heterologous polypeptide, when the latter is a periplasmic or secreted protein. This was for instance the case in the display of CtxB, a periplasmic protein, fused to the TU of *Neisseria *IgA protease [60], or for the display of *Alcaligenes faecalis *pseudo-azurin, also a periplasmic protein, fused to the TU of the *Serratia *serine protease [112]. A second approach is the use of the native sequence signal of the passenger domain of the AT being replaced. This was for instance the case in the display of a fragment of a *Mycobacterium tuberculosis *invasin fused to the TU of AIDA-I [113]. As stated above, some AT exhibit peculiarly long signal sequences. Even though the role of the latter is not definitely established, it seems to be necessary for correct translocation, at least for native passenger domains. When using the TU from an AT bearing a long signal sequence it might therefore be necessary to keep such a signal in order to optimize translocation efficiency, but this was not tested. A third approach is the use of the signal sequence of a periplasmic protein unrelated to either the AT used for display or the heterologous polypeptide displayed. A number of such signal sequences have been used, for instance that of CtxB [5,99], PelB [7], or PhoA [101].

#### - Length of the AT sequence used as a translocator

As stated above, the minimal part of the AT required for translocation across the outer membrane is the TU, consisting of the α-helix and the β-barrel. In practice, however, the precise limits of the α-helix have not been structurally determined for any AT but NalP. Therefore, most constructs used for display are probably leaving a few aminoacids of the passenger domain in the final hybrid. This might be of importance, since several studies show that there is a wide variation in the efficacy of secretion depending on the length of the AT passenger domain left in the hybrid construct. In general, the more of the C-terminal portion of the passenger domain is left in the hybrid, the better is the expression level. This was observed with the *Neisseria *IgA protease [39], IcsA [63], AIDA-I [76] and EstA [108]. This was not strictly the case, however, with the *Serratia *serine protease, where a hybrid having a longer portion of the passenger domain was less expressed than a hybrid with a shorter portion [112]. As described above, the C-terminal part of the passenger domain is the autochaperone region which was shown to improve the secretion of native passenger domains [43,45,46]. Since some studies argue that this effect is mediated in part by interactions of the autochaperone domain with the TU [44], keeping the autochaperone domain in the construction of AT-based display vectors might ensure optimal translocation efficiency. Additionally, in some models, the autochaperone folding is thought to provide the energy driving translocation [31], another reason to keep these domains in display constructs. In some studies this issue has been completely averted by displaying proteins as fusion to a full length passenger domain, such as in the case of lipases fused to EstA [101]. Interestingly, the display of the *S. marcescens *lipase (613 aminoacids, making it, to our knowledge, the longest protein displayed by an AT), used this system [101].

#### - Release of the displayed polypeptide

As described above, in some AT the passenger domain stays linked to the outer membrane whereas in others it is cleaved and released in the extracellular milieu. Since in most cases the passenger domain is replaced by the heterologous polypeptide, the release is usually avoided for lack of catalytic and/or cleavage site. However, even when the autocatalytic release of the AT is prevented by a mutation in the catalytic site or a replacement of the original passenger domain by a heterologous polypeptide, residual release can be mediated by the outer membrane protease OmpT or other unknown proteases [61]. Consequently, the use of *ompT*^- ^strains has been shown to increase the expression level of polypeptides displayed by AT [65,108,114]. Conversely, in the case where the protein of interest is to be released, the construct should bear a cleavage site freely accessible and the protease responsible for the cleavage should be provided. This approach is not always successful, however, probably because the cleavage site can easily be masked by the heterologous passenger domain [112].

#### - Host factors

As described above, depending on the presence or absence of host factors such as DsbA or OmpT, a fusion to the TU of an AT can yield vastly different levels of expression [62]. Similarly, the periplasmic proteases DegP and DegQ were shown to degrade fusion partners to the TU of AIDA-I, but the use of *degP*^- ^or *degQ*^- ^strains did not improve the display [64]. Lastly, it should be noted that the periplasmic peptidyl-prolyl isomerase FkpA was found to increase the efficiency of display of a ScFv or an isolated immunoglobulin variable domain fused to the TU of *Neisseria *IgA protease [67]. The influence of more host factors need to be addressed in future studies.

## Conclusion

Using secretion systems or secreted proteins naturally present in bacteria as tools for surface display of foreign polypeptides represents an attractive prospect for many biotechnological applications. However, the complexity of most secretion systems and the structural constraints bearing on secreted proteins can be a disadvantage. By contrast, AT of Gram-negative bacteria stand out with their simplicity and versatility. AT tolerate the replacement of their N-terminal passenger domain by a variety of heterologous proteins, with apparently few constraints on the nature of the polypeptides to be displayed. As our understanding of the molecular mechanism of the biogenesis of AT gets more detailed, however, some new considerations are emerging when considering these proteins as display systems. First, folding of the polypeptide to be displayed in the periplasm prior to translocation can have some important effects on translocation, either because of the size or the shape attained by the heterologous protein. Second, the presence of AT-specific signal sequence and of an autochaperone domain can also have dramatic consequences on translocation. And third, it should be kept in mind that many AT are cleaved after translocation and released in the extracellular milieu. These considerations should not be viewed as limitations of the AT display system. Indeed, AT have already proven to be extremely efficient as carrier proteins and it can be expected that, as our understanding of these proteins grows, we will be able to make the most out of their exploitation. In the future, we can expect many important advances, such as the clarification of the role of the unusually long signal sequences of some AT, a better definition of the translocation mechanism and the evaluation of the influence of periplasmic and outer membrane proteins in the translocation process. This will in turn allow the design of better constructs with all the sequences of the AT that are important for efficient secretion, and the selection of an optimized host.

## Abbreviations

AIDA-I: Adhesin involved in diffuse adherence

AT: Autotransporter

CtxB: Cholera toxin B subunit

FACS: Fluorescence assisted cell sorting

OMP: Outer membrane protein

Sec: General secretion machinery

ScFv: Single chain antibody variable fragment

SPATEs: Serine proteases autotransporters of *Enterobacteriacae *

Tat: Twin arginine translocation

TPS: Two-partner secretion

TU: Translocation Unit

## Competing interests

The author(s) declare that they have no competing interests.

## Authors' contributions

Nancy Rutherford and Michael Mourez have contributed equally to this review.
